# Assessment of UV Protection for Children's Summer Clothing

**DOI:** 10.7759/cureus.44137

**Published:** 2023-08-25

**Authors:** Yonesha Cole, Amber M Ilyas, Erum N Ilyas

**Affiliations:** 1 Dermatology, Drexel University College of Medicine, Philadelphia, USA; 2 Health and Environmental Impact, AmberNoon LLC, King of Prussia, USA

**Keywords:** uv protection, pediatric dermatology, sun protective clothing, upf, sun protection

## Abstract

Introduction: Excess ultraviolet (UV) radiation exposure during childhood poses a particular long-term risk of developing skin cancer later in life however recent studies have called attention to the absorption of chemical sunscreen ingredients into the bloodstream with unclear significance. This has led to recommendations to consider sun protection in the form of clothing to reduce the need for sunscreen products.

Methods: Ten brands of routine summer clothing not labeled as sun protective from five distributors marketed for children were identified with varying price ranges. Summer clothing items consisting of T-shirts and tops were evaluated for fiber composition, cost, and UPF testing was performed to determine UVA and UVB transmittance.

Results: Around 88.2% of blended textile compositions compared to 60% of 100% cotton textiles offered adequate UV protection with an Ultraviolet Protection Factor (UPF) greater than 15. 58% of blended fabrics compared to 50% of cotton textiles offered a UPF greater than 30. There was no correlation between brand and cost with UPF values.

Conclusions: In the absence of regulations for testing and labeling of all children’s garments for UV protection, guidelines for the pediatric population for choosing clothing for the summer should reflect the information available to consumers that is easy to understand and more likely to result in higher UPF values. Based on fiber composition, blended textiles are more likely to have adequate UV protection compared to 100% cotton or 100% polyester.

## Introduction

Ultraviolet (UV) radiation exposure poses a risk to overall skin health via potential damage to the DNA of cells. Excess UV exposure during childhood poses a particular long-term risk of developing skin cancer later in life [1]. The earliest campaigns for childhood sun protection behavior were based on a study in 1986 by Stern et al. that estimated 78% of cumulative lifetime UV exposure occurs in children and teenagers as a result of outdoor summertime activity and that adequate protection early in life should reduce the long term risk of skin cancer [2]. Subsequent studies have demonstrated that UV exposure to children may be somewhat comparable to adults [3]. Thieden et al. demonstrated that 20% of cumulative lifetime UV exposure occurs before the age of 20 [4]. Wide variability will exist based on lifestyle, occupation, and region within the United States. A study by Wu et al. suggests that a history of sunburns may have a higher association with melanoma development as compared to non-melanoma skin cancers, such as basal cell carcinoma and squamous cell carcinoma, which appear to be related more to chronic UV exposure [5].

A study by Harrison et al. demonstrated that children who wear sun-protective clothing developed fewer new nevi on clothing-protected areas of the skin. This study suggested that UV exposure plays a role in the development of new nevi and a reduction in new nevus development through the use of sun-protective clothing has the potential to reduce the long-term risk of melanoma [6]. A study by Haenssle et al. found over 54% of melanomas developed in preexisting nevi [7].

Adequate UV protection balanced with the need for vitamin D production during childhood requires a comprehensive approach to options with regard to skin protection. With studies demonstrating the absorption of chemical sunscreen additives with unclear health implications [8], options for sun protection in clothing have been increasingly recommended by the American Academy of Pediatrics (AAP). Specifically, the AAP recommends “select clothes made of tightly woven fabrics. Cotton clothing is both cool and protective” [9]. A study by Gambichler et al. demonstrated in an investigation of 236 summer textiles in 2001 that a third of textiles studied did not offer adequate UV protection as defined as an ultraviolet protection factor (UPF) less than 15 with only half with a UPF rating of 30+ [10]. There is no current standard minimum UPF requirement for summer textiles.

A survey by the American Academy of Dermatology (AAD) found that 74% of parents demonstrate concern for their children’s sun protection compared to their parents. Amongst other sun protective measures, recommendations include “dress kids in sun-protective clothing, such as a lightweight and long-sleeved shirt, pants…” [11] Although “sun-protective clothing” is referenced, it appears to be defined as “lightweight and long-sleeved shirt, pants” without providing details on textiles to choose and UPF value to seek. 

The UPF scale is used to assess the UV-blocking ability of textiles. The UPF rating is based on both UVA and UVB blockage of a textile with ratings assigned based on the level of protection offered. A UPF rating of 15 corresponds to textiles that block 93.3% of UV radiation transmittance, a UPF of 30 blocks 96.7% of UV radiation transmittance, and a UPF of 50+ blocks over 98% of UV radiation transmittance. The Skin Cancer Foundation recommends that fabric have a UPF of at least 30 to qualify for their seal of recommendation [12]. It is also important to note that a UPF rating of 15-24 is considered “good,” 25-39 is “very good,” and 40-50+ is “excellent” [13].

The American Society for Testing Materials (ASTM), has assigned standard practice to label garments with UPF values with protocols that designate the UPF value assigned to a garment as the lowest UPF value obtained after the garment is subjected to 40 launderings to simulate “consumer use for two years” [14]. For textiles manufactured via embedding inorganic sunscreen filters into the textile or treated with organic sunscreen filters to increase the UV protective qualities of the textile, there is the potential for these finishes to be affected by the laundering process. Fernau et al. demonstrated that for textiles not treated with organic or inorganic sunscreen filters, the UPF ratings of untreated textiles remained relatively stable through 50 laundering cycles [15].

To assess the baseline UV protection offered by children’s summer clothing found at varying price ranges upon purchase, this study was undertaken to review several popular brand names available for clothing options to determine how effective routine children’s summer clothing is for protecting against UV exposure based on fiber composition, store, and cost.

## Materials and methods

It was determined that the study did not involve the use of human subjects and IRB approval was not necessary. Ten brands of clothing from five stores marketed for children were identified of varying price ranges; including Crewcuts (J. Crew; New York), Cat & Jack (Target; Minneapolis, Minnesota), CoComelon (Distributed through Target; Irvine, California), Disney (Distributed through Target; Glendale, California), Nike (Distributed through Dick’s Sporting Goods; Beaverton, Oregon), Adidas (Distributed through Dick’s Sporting Goods; Herzogenaurach, Germany), Under Armour (Distributed through Dick’s Sporting Goods; Baltimore, Maryland), Old Navy (San Francisco, California), Garanimals (Distributed through Walmart; New York, New York), and GapKids (Gap, San Francisco, California). Garments were purchased during the first week of June 2023 with items specifically marketed as summer clothing items. Routine summer clothing, consisting of T-shirts and dresses, was chosen. Only white or lightest colored clothing options were chosen to control for the role of potential dyes and pigments added to clothing that could impact the UPF values. Gambichler et al. demonstrated that black or darkly colored textiles often have a UPF of 30+ compared to white textiles [8]. Different styles were chosen by each brand when available to consider the possibility of different textiles used within brands that may still have similar fiber composition and potentially different construction to determine if this may impact the UPF value. All clothing items were children’s items sized for toddlers up to children's size 7.

These garments were tested upon purchase for UPF with the UV 2000F Ultraviolet Transmittance Analyzer (LabSphere, North Sutton, NH). The LabSphere UV 2000F analyzer measures UV transmittance in the ultraviolet wavelength region from 250 to 450 nm to determine UPF values. During testing, UVA and UVB rays via a Xenon Flash Lamp emitting a UV dose of < 0.2 J/cm^2^ are passed five times in different directions throughout the fabric and provide an average UPF measurement based on the AATCC 183:2004 protocol. The AATCC 183:2004 is considered the standard test protocol for the United States to determine the UPF of textiles [16]. Although labeling for UPF requires determining the lowest UPF values after 40 laundering cycles to simulate routine wear and tear, our assessment was only focused on the initial rating obtained on purchase and not over routine wear and tear. The maximum UPF assignable by the LabSphere UV 2000F analyzer is 2000 indicating the percent of UVA and UVB blockage as 99.5%.

The store, fiber composition of each garment, UPF rating, percent UVA blocking, and percent UVB blocking were determined and documented. The cost of each garment was also noted. Basic descriptive statistics were used as well as determining the correlation between UPF values and fiber composition, cost, and store using the Pearson correlation coefficient to determine the r value for comparison purposes.

## Results

A total of 31 garments were tested, 28 T-shirt styles and three summer dresses. The overall results of UPF testing for summer children's T-shirt styles and dresses are found in Table 1 as listed by store, brand, style, fiber composition, price, UPF, and UVA and UVB percentage blocked by the textile.

**Table 1:** Summer T-shirt styles tested by store, brand, composition, cost, UPF value, UVA and UVB transmittance blockage. Variations in T-shirt styles available by the store were designated by assigning an alpha character (A through F) followed by another letter to designate the store of purchase. UPF: Ultraviolet protection factor; UVA and UVB: Ultraviolet A and Ultraviolet B radiations

Store	Garment Brand	Style	Composition	Price	UPF	UVA % Blocking	UVB % Blocking
J Crew	Crewcuts	T-shirt Style AJ	100% Cotton	$24.50	29	97.26%	96.47%
J Crew	Crewcuts	T-shirt Style BJ	100% Cotton	$24.50	158	99.10%	99.29%
J Crew	Crewcuts	T-shirt Style CJ	100% Cotton	$32.50	62	97.09%	98.21%
J Crew	Crewcuts	T-shirt Style DJ	100% Cotton	$34.50	8	82.31%	89.96%
Target	Cat & Jack	T-shirt Style AT	60% Cotton; 40% Recycled Polyester	$4.00	34	95.92%	97.44%
Target	Cat & Jack	T-shirt Style BT	60% Cotton; 40% Recycled Polyester	$5.00	24	87.70%	96.84%
Target	CoComelon	T-shirt Style CT	100% Cotton	$8.00	13	85.65%	94.06%
Target	Disney	T-shirt Style DT	53% Cotton; 47% Polyester	$8.00	33	94.22%	97.63%
Dick's Sporting Goods	Nike	T-shirt Style AD	60% Cotton; 40% Polyester	$22.00	39	97.30%	97.64%
Dick's Sporting Goods	Nike	T-shirt Style BD	100% Polyester	$25.00	11	84.90%	92.42%
Dick's Sporting Goods	Adidas	T-shirt Style CD	100% Cotton	$25.00	36	95.83%	97.58%
Dick's Sporting Goods	Adidas	T-shirt Style DD	95% Cotton; 5% Spandex	$22.00	9	90.04%	90.13%
Dick's Sporting Goods	Under Armour	T-shirt Style ED	60% Cotton; 40% Polyester	$25.00	22	94.52%	95.93%
Old Navy	Old Navy	T-shirt Style AO	57% Cotton; 38% Polyester; 5% Spandex	$14.99	66	92.40%	99.34%
Old Navy	Old Navy	T-shirt Style BO	60% Cotton; 40% Polyester	$9.99	42	97.07%	97.96%
Old Navy	Old Navy	T-shirt Style CO	62% Polyester; 33% Rayon; 5% Spandex	$12.99	80	97.55%	99.00%
Old Navy	Old Navy	T-shirt Style DO	50% Cotton; 50% Polyester	$9.99	25	87.26%	97.30%
Old Navy	Old Navy	T-shirt Style EO	60% Cotton; 40% Polyester	$9.99	70	97.08%	98.97%
Old Navy	Old Navy	T-shirt Style FO	100% Cotton	$19.99	5	76.22%	81.60%
Walmart	Garanimals	T-shirt Style AW	60% Cotton; 40% Polyester	$5.98	92	98.81%	99.08%
Walmart	Garanimals	T-shirt Style BW	60% Cotton; 40% Polyester	$5.98	36	90.21%	98.27%
Walmart	Garanimals	T-shirt Style CW	60% Cotton; 40% Polyester	$3.98	45	96.98%	98.02%
Walmart	Garanimals	T-shirt Style DW	100% Cotton	$8.98	52	98.54%	98.41%
Gap Kids	Gap Kids	T-shirt Style AG	60% Cotton; 40% Polyester	$24.95	43	94.11%	98.25%
Gap Kids	Gap Kids	T-shirt Style BG	100% Cotton	$24.95	10	83.68%	91.75%
Gap Kids	Gap Kids	T shirt Style CG	52% Cotton; 29% Polyester; 15% Rayon; 4% Spandex	$24.95	24	88.10%	97.25%
Gap Kids	Gap Kids	T shirt Style DG	100% Cotton	$19.95	49	98.70%	98.19%
Gap Kids	Gap Kids	T shirt Style EG	55% Linen; 45% Cotton	$29.95	10	89.81%	90.47%

Of the 28 summer T-shirt styles tested, six stores and 10 brands were represented. Ten of the 28 summer T-shirt styles tested were 100% cotton, one was 100% polyester, and the remaining 17 were blended fiber textiles. 

Limiting the results to only garments composed of 100% cotton, ten T-shirt styles from six brands and six stores were evaluated. The UPF measurements vary from 5 to 158, ranging in price from $8 to $34.50. Of these cotton garments, only five (50%) offered a UPF value of 30+ with four (40%) offering inadequate UV protection with a UPF less than 15. Pearson’s Correlation Coefficient was used to determine if there was a correlation between cost and UPF value with a negligible correlation of 0.08 found for 100% cotton T-shirts.

Of the 28 summer T-shirt styles tested, 17 were composed of blends of cotton/spandex, linen/cotton, cotton/polyester, cotton/polyester/rayon/spandex, cotton/recycled polyester, cotton/polyester/spandex, and polyester/rayon/spandex ranging in price from $3.98 to $29.95. The UPF measurements varied from 9 to 92. Of these 17 garments tested, two out of 17 (11.8%) did not have adequate UV protection with a UPF less than 15 while 11 of 17 (65%) garments offered a UPF of 30+. Evaluating all 17 blended fiber summer T-shirt styles, a low negative correlation coefficient of -0.41 was noted between cost and UPF value. One garment was made of 100% polyester and did not offer adequate UPF at a value of 11.

Evaluating the T-shirts with cotton in the composition, 26 T-shirts contained a component of cotton ranging from 45% to 100%. The UPF values ranged from 5 to 158 and the price ranged from $3.98 to $34.50. A negligible correlation coefficient of 0.03 was found between the percentage of cotton and the UPF value of the garment. For T-shirts with polyester in the composition, 16 garments contained a component of polyester ranging from 29% to 100% with UPF values ranging from 11 to 92 and a price range from $3.98 to $25. A negligible correlation coefficient was found of -0.2 between the percent of polyester and the UPF value of the garment. Evaluating the correlation between the percentage of polyester and the percent UVA blocking and UVB blocking it was determined that a correlation coefficient of -0.39 and -0.71 exists indicating a low negative and high negative correlation between the percent of polyester and percent UVA and percent UVB blocking respectively. For cotton, a -0.13 and -0.34 correlation coefficient was found between the percent of cotton and the percent UVA and UVB blocking, respectively, by the garment indicating a negligible correlation between cotton composition and UVA blocking and a low negative correlation to UVB blocking. 

Every garment tested contained a component of cotton and/or polyester. Given the negative correlation noted between the percent of cotton and polyester concentration on the UVB blocking ability of the garment, limiting the sample tested to summer T-shirts with mixed blends with polyester composition ranging from 29% to 62% and cotton composition ranging from 45% to 60%, there was a UPF range from 10 to 92 in the 16 summer T-shirt styles that met these criteria. One of these garments had an inadequate UPF value of less than 15 (6.3%) and 10 (62.5%) garments had a UPF of 30+. A moderate positive correlation between the percent cotton and UPF was noted with a correlation coefficient of 0.50 and a low positive correlation coefficient of 0.30 between the percent of polyester. A low negative correlation of -0.36 was noted between the UPF value and cost for these garments.

For the summer dresses tested, each garment comprised two layers of fabric. For each garment tested, adding a second layer of fabric increased the overall UPF value of each garment tested regardless of each layer’s fiber composition. However one garment, in spite of two layers, still failed to reach a UPF of 30+. A correlation coefficient of -0.11 was found between cost and UPF indicating a negligible association between these two values (Table 2).

**Table 2:** Summer children's dress styles tested by brand, composition, cost, UPF value, UVA transmittance blockage, and UVB transmittance blockage. UPF: Ultraviolet protection factor; UVA and UVB: Ultraviolet A and Ultraviolet B radiations

Garment Brand | Style	Style	Composition	Price	UPF	UVA % Blocking	UVB % Blocking
J Crew (Crewcuts)	Dress	Layer 1: 60% Linen; 40% Cotton	$45.00	8	89.21%	88.55%
		Both Layers: 60% Linen; 40% Cotton		49	98.40%	98.40%
Target (Cat & Jack)	Dress	Layer 1: 100% Cotton	$18.00	12	84.91%	90.69%
		Both Layers: 100% Cotton		21	92.44%	96.49%
Walmart (Garanimals)	Dress	96% Polyester; 4% other Fibers	$9.98	6	86.66%	84.35%
		Both Layers: Layer 1 + 100% Cotton		68	98.05%	98.84%

Assigning a numeric value between 1 through 6 to each store with the value assigned based on the average cost of garments with 1 indicating the lowest average cost and 6 indicating the highest average cost per garment, there was no correlation noted between store and UPF value with a correlation coefficient of -0.02 (Table 3).

**Table 3:** Average cost of clothing items at each store.

Store	Average Price	Numeric value assigned to each store
Walmart	$6.23	1
Target	$6.25	2
Old Navy	$12.99	3
Dick's Sporting Goods	$23.80	4
Gap Kids	$24.95	5
J Crew	$29	6

Overall, out of 31 total garments tested, 18 (58%) offered UPF of 30+ with no correlation noted to cost, fiber type, and store. For the layered garments tested, adding a second layer did increase the overall UPF of the garment, however, this did not consistently lead to a UPF over 30. 22.6% of the garments tested did not offer adequate UV protection, defined by a UPF as less than 15. Every garment tested contained a component of cotton and/or polyester. A moderate positive correlation was noted between summer T-shirt styles and UPF with a cotton composition ranging from 45% to 60% and a low positive correlation between summer T-shirt styles and UPF with a polyester composition ranging from 29% to 62% and a low negative correlation between UPF and cost for these garments.

## Discussion

Clothing as a sun protective measure for children can offer a balance of UV exposure and protection. Balancing the detrimental effects of UV exposure with the benefits of vitamin D production is essential in children in particular. Clothing can reduce the amount of UV transmitted to the skin needed for pre-vitamin D3 synthesis, however, the amount of UV protection offered by clothing can vary based on the textiles used [17]. Although sunscreen use alone has not been associated with compromised vitamin D production, the use of sun-protective clothing along with other sun protection behaviors such as seeking shade can impact vitamin D levels [18]. However, exposure to the arms and legs for less than 30 minutes twice weekly during midday hours is thought to be sufficient for vitamin D production [19]. UV protection via clothing can be effective while reducing the impact on vitamin D production if guidelines clearly state methods to achieve benefits balanced with risks. Based on a 2001 study by Gambichler et al., a third of summer textiles not limited to children’s clothing studied at that time did not offer adequate UV protection with only half with a UPF rating of 30+ concluding that summer fabrics should be measured and labeled to assist consumers in choosing garments to support their sun protection needs [10]. 

Several studies have demonstrated the UPF of standard clothing tends to be influenced by fiber type, color, and construction. The cover factor is determined by the tightness of the weave or how close the fibers are drawn together during textile construction and is considered one of the most important factors in determining the UPF of routine textiles [20,21]. For the average consumer shopping for clothing that may offer added UV protection, the store the garment is purchased from, the style of the garment, cost, color, and fiber composition are the features readily apparent for consumers to consider. The construction of the garment and the weight of the fabrics are not typically referenced on product labels.

The results of this study are limited to children’s summer clothing. The results for UPF values of summer textiles based on summer children’s clothing items purchased in June of 2023 revealed that 22.6% did not offer adequate UV protection with a UPF less than 15 while 58% offered a UPF of 30+. This indicates that there was a decrease in the percentage of summer clothing items offering what would be considered inadequate UV protection from about 33% in 2001 to 22.6% in 2023. There was also an increase in the percentage of clothing items with a UPF of 30+ from 50% in 2001 to 58% in 2023. Although a change has been noted, there are still limited options easily available for consumers to find for children seeking sun protection without resorting to the use of specialty brands dedicated to sun-protective clothing. 

The AAP recommendations ask consumers to seek “clothes made of tightly woven fabrics” while stating “cotton clothing is both cool and protective”. The recommendation does not define “tightly woven” to assist consumers in navigating choices [9]. Without a clear definition for this terminology, the tightness of the weave of a fabric can be somewhat subjective to the average consumer. In purchasing clothing, consumers can objectively identify store, style, color, cost, and fiber composition for clothing. Based on the criteria for summer clothing for children recommended by the AAP, the only factor recommended to consider in purchasing summer clothing that is available for consumers to use is the fiber composition of cotton. Seeking 100% cotton clothing as the fiber of choice we found only 50% of garments tested offered adequate UV protection while 40% of cotton garments did not offer adequate UV protection with a UPF of less than 15. These findings suggest that the guidance offered for sun protection for children from common clothing brands may not provide the best options available to provide adequate UV protection. Without a clear definition for “tightly woven” along with the lack of labeling details to help guide consumers on the cover factor of the textiles in the garments they choose, this recommendation may be difficult for consumers to adapt into practice. The recommendation for cotton clothing may not provide the best protection for consumers seeking to provide children with adequate UV protection. These findings are consistent with prior studies that have demonstrated that fabric construction of a textile plays an important role in determining the UV protection offered [22]. However, the average consumer does not have access to data on the factors associated with construction aside from fiber composition. 

The AAD recommendations reference “lightweight” garments and “long-sleeved”. Lightweight textiles for the summer tend to be composed of cotton and/or linen. However, a clear definition for the term “lightweight” is not offered and garments are not typically labeled by the weight of their respective textiles. The recommendation for “long-sleeved” garments references the style of the garment without consideration of the fiber type chosen. Consideration of the body surface area covered by clothing is an important criterion to integrate in recommendations for choosing clothing for UV protection. Practically speaking, the only way that clothing can protect the skin against UV exposure is by physically covering the skin. Downs et al. recommend manufacturers consider including the garment protection factor, or GPF, on labeling for sun-protective clothing and potentially create standards for the amount of body surface area covered [23]. However, without clear specifications on fiber composition and/or UPF values for sun-protective clothing to choose from, consumers have little guidance in choosing effective options. For example, choosing a linen/cotton blend with long sleeves based on the UPF values obtained in this study could potentially provide a UPF of 10 to the covered areas, leaving these areas with inadequate UV protection. 

Based on testing on garments with blended fiber compositions, 11.8% did not have adequate UV protection with a UPF of less than 15. The only garment tested with 100% polyester was found to have inadequate UV protection with a UPF of 11. With 65% of these garments offering a UPF of 30+ and 88.2% of garments offering a UPF of greater than 15, choosing a garment with a blended fiber composition appears to offer better UV protection compared to 100% cotton or 100% polyester. More so, textiles with blended compositions with cotton ranging from 45% to 60% and/or polyester ranging from 29% to 62% demonstrated a moderate correlation between cotton and UPF and a low positive correlation between polyester and UPF value. These compositions appear to indicate a more “balanced” fiber profile as a better indicator of adequate UPF offered by a garment. Seeking garments made of blended compositions containing cotton and/or polyester, ideally with more balanced composition blends ranging from 45% to 60% for cotton and/or polyester ranging from 29% to 62%, consumers may have a higher likelihood of choosing a garment with a higher UPF value.

Of note, summer garments made of lighter-weight textiles may have two layers of the same or varying composition. Logically, it may be assumed that adding a second layer will naturally increase the UV protection offered by the final garment. The data obtained from two layered garments revealed that the UPF of the final garment consistently increased upon adding the second layer compared to the first, however, adding a second layer did not consistently raise the UPF over 30. 

Store name and cost did not consistently correlate with higher UPF values. Interestingly, when limiting the evaluation to summer T-shirt styles made of balanced blended compositions, a low negative correlation was noted between cost and UPF value suggesting that spending more money does not result in increased UV protection from the garment. 

In consideration of factors that consumers with an interest in UV protection can readily review in choosing garments for children, fiber composition could be considered followed by price if not choosing clothing specifically labeled with a UPF rating. Recommending styles that cover more body surface area such as long sleeves and pants will maximize the protection offered by clothing alone.

There are several limitations to note for this study. The first limitation is the number of garments tested. In seeking garments to test, there were limited options commercially available in terms of compositions and styles to compare and limited offerings by stores for children's clothing. The color white or lightly colored garments were intentionally chosen from each style available to compare values based on composition and reduce the impact of pigmentation on UPF values. Clothing dyes can increase the UPF value independent of the composition and construction of the textile although not as significantly as the construction of the textile alone [24]. Another limitation to note is that an initial UPF value was obtained upon purchase of the garment and not after washing. Although consumers may wash clothing upon purchase prior to wearing, Fernau et al. demonstrated that clothing not specifically treated with UV chemical finishes was more likely to retain stable UPF values through laundering [15].

## Conclusions

Recognition of the need for adequate UV protection for children that is safe and effective along with addressing consumer concerns about the safety of sunscreen products has led to an increasing focus on clothing as an option for UV protection. Although there are brands dedicated to offering sun-protective clothing, routine children’s summer clothing is not tested for UV protection. For consumers seeking routine clothing, general recommendations on products to choose from are intended to help guide effective choices. Without testing and labeling for all garments, consumers seeking options for UV protection may rely on recommendations from the medical community to guide ideal clothing choices. Current guidelines for the pediatric population reference cotton clothing and lightweight textiles. The findings of this study suggest that garments with balanced blended fiber compositions consisting of cotton and/or polyester are more likely to offer adequate UV protection for children. Consumers should factor in the style of clothing to consider covering more body surface area to maximize the protection offered by clothing alone.

## References

[1] Volkmer B, Greinert R (2011). UV and children's skin. Prog Biophys Mol Biol.

[2] Stern RS, Weinstein MC, Baker SG (1986). Risk reduction for nonmelanoma skin cancer with childhood sunscreen use. Arch Dermatol.

[3] Godar DE (2001). UV doses of American children and adolescents. Photochem Photobiol.

[4] Thieden E, Philipsen PA, Sandby-Møller J (2004). Proportion of lifetime UV dose received by children, teenagers and adults based on time-stamped personal dosimetry. J Invest Dermatol.

[5] Wu S, Cho E, Li WQ (20161). History of severe sunburn and risk of skin cancer among women and men in 2 prospective cohort studies. Am J Epidemiol.

[6] Harrison SL, Buettner PG, Nowak MJ (2023). Sun-protective clothing worn regularly during early childhood reduces the number of new melanocytic nevi: the North Queensland sun-safe clothing cluster randomized controlled trial. Cancers (Basel.

[7] Haenssle HA, Mograby N, Ngassa A (2016). Association of patient risk factors and frequency of nevus-associated cutaneous melanomas. JAMA Dermatol.

[8] Matta MK, Florian J, Zusterzeel R (2020). Effect of sunscreen application on plasma concentration of sunscreen active ingredients: a randomized clinical trial. JAMA.

[9] (2023). American Academy of Pediatrics: Top Safety Tips for Preventing Heat, Sun-Related Illnesses in Children this Summer. https://www.aap.org/en/news-room/news-releases/health--safety-tips/american-academy-of-pediatrics-top-safety-tips-for-preventing-heat-sun-related-illnesses-in-children-this-summer/.

[10] Gambichler T, Rotterdam S, Altmeyer P (20011). Protection against ultraviolet radiation by commercial summer clothing: need for standardised testing and labelling. BMC Dermatol.

[11] (2020). New AAD survey: 74% of parents today say they worry about sun protection more with their children than their parents did with them. https://www.aad.org/news/survey-parents-worry-about-sun-protection-more-with-their-children.

[12] (2019). Beyond Sunscreen: UPF Clothing Provides Excellent Fall Sun Protection. https://www.skincancer.org/press/beyond-sunscreen-upf-clothing-sun-protection/.

[13] (2019). Standard Specification for Labeling of UV-Protective Textiles. https://www.astm.org/d6603-19.html.

[14] (2023). Standard Practice for Preparation of Textiles Prior to Ultraviolet (UV) Transmission Testing. American Society for Testing and Materials. https://www.astm.org/d6544-21.html.

[15] Fernau E, Ilyas SM, Ilyas EN (2023). The impact of routine laundering on ultraviolet protection factor (UPF) values for commercially available sun-protective clothing. Cureus.

[16] (2020). TM183-TM 183 transmittance or blocking of UV radiation through fabric. https://members.aatcc.org/store/tm183/579/.

[17] Parisi AV, Wilson CA (2005). Pre-vitamin D effective ultraviolet transmission through clothing during simulated wear. Photodermatol Photoimmunol Photomed.

[18] Passeron T, Bouillon R, Callender V (2019). Sunscreen photoprotection and vitamin D status. Br J Dermatol.

[19] Pela I (2012). How much vitamin D for children?. Clin Cases Miner Bone Metab.

[20] Aguilera J, de Gálvez MV, Sánchez-Roldán C (2014). New advances in protection against solar ultraviolet radiation in textiles for summer clothing. Photochem Photobiol.

[21] Kostajnšek Kostajnšek, K.; Dimitrovski, K K (2021). Use of extended cover factor theory in UV protection of woven fabric. Polymers.

[22] Reinert G, Fuso F, Hilfiker R, Schmidt E (1997). UV-protecting properties of textile fabrics and their improvement. Textile Chemist & Colorist.

[23] Downs NJ, Harrison SL (2018). A comprehensive approach to evaluating and classifying sun-protective clothing. Br J Dermatol.

[24] Kan CW, Au CH (2015). In-vitro analysis of the effect of constructional parameters and dye class on the uv protection property of cotton knitted fabrics. PLoS One.

